# Effects of Insulin-like Growth Factor I and Follicular Fluid on In Vitro Growth of Cultured Oocytes

**DOI:** 10.3390/biology15010046

**Published:** 2025-12-26

**Authors:** Yunfei Diao, Dengrong Zhai, Yunsu Wu, Puyuan Ai, Shuxuan Liu, Xiaoxia Li

**Affiliations:** 1 College of Animal Science and Technology, Jilin Agricultural Science and Technology College, Jilin 132109, China; diaoyunfei888@jlnku.edu.cn (Y.D.); zdr201314@yeah.net (D.Z.); 13082266735@163.com (Y.W.); 18241300848@163.com (P.A.); 13039075335@163.com (S.L.); 2 Jilin Inter-Regional Cooperation Centre for the Scintific and Technological Innovtion of Ruminant Precision Nutrition and Smart and Ecological Farming, Jilin 132109, China; 3 College of Agricultural, Yanbian University, Yanji 133000, China

**Keywords:** oocyte–granulosa cell complexes, IGF-I, follicular fluid, pig, apoptosis

## Abstract

Oocyte-granulosa cell complexes (OGCs) cultivation is crucial for advancing reproductive biotechnology but remains incomplete and needs further optimization. Insulin-like growth factor-I (IGF-I) regulates granulosa cell proliferation and apoptosis, and numerous studies have confirmed its role in promoting ovarian follicle development. Porcine follicular fluid (PFF) contains factors beneficial for oocyte growth, which may enhance oocyte development. To investigate whether IGF-I and PFF improve the in vitro culture efficiency of porcine OGCs, we cultured OGCs with IGF-I (0, 10, 50, 100 ng/mL) and PFF (from 3 to 6 mm follicles) at concentrations of 0, 2.5%, 5%, 10%, respectively. The results revealed that 50 and 100 ng/mL IGF-I significantly increased the antrum formation rate of OGCs (from 61.11 ± 7.35% to 88.89 ± 7.35%) and diameter growth of oocytes (from 108.77 ± 0.27 µm to 114.94 ± 0.58 and 113.29 ± 0.50 µm, respectively). However, only the 50 ng/mL group, but not the 100 ng/mL group, significantly improved the maturation rate (38.13 ± 3.77% vs. 25.00 ± 3.27%, *p* < 0.05) of oocytes. Additionally, 50 ng/mL IGF-I downregulated *BAX* (a pro-apoptotic gene) and upregulated *BCL-2* (an anti-apoptotic factor) in granulosa cells, ultimately reducing apoptosis. In contrast, none of the PFF doses used in this study induced the formation of enclosed antrum-like structures in OGCs, nor did they significantly enhance their in vitro development. Our findings demonstrate that 50 ng/mL IGF-I effectively promotes the in vitro growth of porcine early antral follicle-derived OGCs by reducing apoptosis, whereas tested PFF concentrations had no beneficial effects and induced abnormal granulosa cell growth. How PFF modulates the adherent and spreading growth of granulosa cells has not been fully elucidated and requires further clarification.

## Simple Summary

In vitro culture of oocyte granulosa cell complexes (OGCs) is one approach to obtain large quantities of usable oocytes, supporting assisted reproductive technologies, yet the current technical system remains incomplete. This study aimed to explore whether insulin-like growth factor I (IGF-I) and porcine follicular fluid (PFF) could improve porcine OGCs in vitro culture efficiency. Results showed that 50 ng/mL IGF-I significantly increased OGCs’ antrum-like structure formation rate and oocyte growth rate; it also reduced granulosa cell apoptosis by decreasing pro-apoptotic gene *BAX* expression and increasing anti-apoptotic factor *BCL-2* expression. In contrast, the different concentrations of PFF used in this study (2.5%, 5% and 10%, *v*/*v*) caused abnormal morphology of OGCs and failed to promote oocyte in vitro development. We conclude that 50 ng/mL IGF-I effectively promotes in vitro oocyte growth. This finding provides data support for optimizing reproductive technologies, obtaining large quantities of oocytes, and conserving germplasm resources of livestock or endangered animals.

## 1. Introduction

With the rapid advancement of biotechnologies—such as in vitro fertilization, animal cloning, and genome editing—there has been a surge in demand for large quantities of fertilizable oocytes [1]. Despite these technological advances, the large-scale acquisition of high-quality oocytes remains a critical technical bottleneck that demands urgent breakthroughs.

Mammalian ovaries harbor a vast reservoir of oocytes, which are sequestered within diverse types of follicles. Among this extensive pool, only a minute fraction of oocytes are ovulated from mature follicles; in contrast, the overwhelming majority undergo atresia and subsequent apoptosis. The porcine oocytes used for in vitro maturation, fertilization, or other biotechnological studies are usually from large-diameter antral follicles (3~8 mm) [2,3]. This type of follicle is relatively few in number. If large numbers of oocytes from pre-maturation follicles could be effectively utilized, they could not only produce more embryos for livestock production but also be used for the preservation and study of endangered animals. Recently, research has focused on creating in vitro culture (IVC) methods for immature oocytes housed in early antral follicles (EAFs) [4,5,6]. In this approach, oocyte–granulosa cell complexes (OGCs) are isolated from EAFs and cultured in vitro to obtain large numbers of usable oocytes. This strategy has been investigated in mice, cattle, sheep, and pigs [7,8,9,10], with the obtained oocytes already applied to fields such as in vitro fertilization and somatic cell cloning [11,12]. The application of in vitro OGCs culture technology not only alleviates the scarcity of oocytes but also enables the establishment of an in vitro oocyte growth model, serving as an effective technical tool to investigate the mechanisms of oogenesis. However, current in vitro growth systems for early antral follicle-derived OGCs face limitations including low antrum formation efficiency, unstable oocyte survival rate, and poor maturation competence.

Notably, these limitations are closely associated with follicular atresia—a key barrier to OGCs in vitro development—since antrum formation, oocyte growth, and maturation competence all depend on the functional integrity of granulosa cells [13]. Accumulating evidence suggests that granulosa cell apoptosis is a major driver of such atresia in follicles [14,15,16,17], which may directly contribute to the suboptimal outcomes of current OGCs in vitro culture systems. Insulin-like growth factor I (IGF-I) acts as the primary mediator of the IGF system functions. It is widely distributed across animal cells and tissues. In the ovary, IGF-I is secreted by both granulosa cells and theca cells [18]. In mammals, IGF-I serves as a key regulator of ovarian function, affecting granulosa cells, driving the development of terminal follicles and facilitating estradiol production [19,20]. Throughout follicular development, IGF-I also plays a critical regulatory role in promoting granulosa cell proliferation and inhibiting apoptosis [21,22,23], processes that, in turn, support follicular growth [24] and enhance follicular enlargement [25]. Notably, research has confirmed that IGF-I levels are notably higher in healthy follicles than in degenerating follicles; concurrently, the expression of IGF receptors is reduced in atretic follicles [26]. These observations suggest that IGF-I may modulate oocyte development by regulating granulosa apoptosis. However, whether IGF-I can enhance the in vitro growth and development of oocytes obtained from EAFs by reducing granulosa cell apoptosis remains unclear.

In addition, follicular fluid (FF), composed of ovarian secretions and plasma exudates, serves as the microenvironment for oocyte growth and development, and exerts a pivotal regulatory effect on follicular development [27]. FF is a physiologically relevant microenvironment enriched with bioactive molecules—including insulin-like growth factor-I (IGF-I), growth hormone (GH), epidermal growth factor (EGF), growth differentiation factor 8 (GDF8) and extracellular matrix proteins—that directly modulate oocyte development and maturation, as well as the developmental competence of subsequent fertilization embryos [28,29,30,31,32]. A multitude of studies have shown that certain substances in follicular fluid can reflect oocyte quality, fertilization capacity, and subsequent embryonic developmental potential [33,34,35].

Despite the known role of IGF-I in follicular development, its optimal concentration and mechanism of action in the culture of porcine early antral follicle-derived OGCs remain unclear. Additionally, most studies have reported the effects of porcine follicular fluid (PFF), focusing on oocyte maturation in oocytes derived from 3 to 8 mm follicles [36,37]. The effects of PFF on the growth and development of oocytes derived from early antral follicles have rarely been reported. This study aims to fill these gaps by investigating the dose-dependent effects and mechanism of IGF-I, as well as the role of PFF, in the in vitro growth of porcine OGCs. Our research focuses on the in vitro culture of porcine early antral follicle-derived OGCs, a model that has received less attention in previous IGF-I studies. To optimize the OGC culture system, this study examined the effects of different concentrations of IGF-I or PFF on OGC growth in vitro. The expression of apoptotic and antiapoptotic genes in OGCs cultured in vitro has also been detected.

## 2. Materials and Methods

### 2.1. Ethics Statement

The experimental protocols described hereinafter were reviewed and approved by the Institutional Animal Care and Use Committee (IACUC) of Jilin Agriculture Science and Technology College, and all procedures were performed in strict adherence to the committee’s guidelines. The approved research protocol identifier is LLSC202402030.

### 2.2. Experiment Design

#### 2.2.1. Preliminary Experiment: Prescreening of OGC Culture Duration and Preliminary Screening Concentrations of IGF-I and PFF

In the preliminary experiment, OGCs were cultured for 10–16 days and treated with different concentrations of IGF-I (0, 10, 50, 100, 200, and 500 ng/mL) or PFF (0, 2.5%, 5% and 10%; *v*/*v*) to prescreen the appropriate culture time and concentration range for the experiment.

#### 2.2.2. Experiment 1: Screening of Optimal IGF-I Supplemental Concentration

To determine the optimal supplemental concentration of IGF-I, different concentrations of IGF-I (0, 10, 50, and 100 ng/mL) were added to the culture system. The effects of these different concentrations on antrum-like structure formation of OGCs, oocyte growth, and oocyte viability (including oocyte survival and maturation) were detected and analyzed. To investigate the effect of IGF-I on the growth of OGCs, 335 OGCs were allocated to 9 experimental replicates, and oocyte diameter, antrum formation rate, and the onset time of antrum formation were measured. Separately, to evaluate the effect of IGF-I on the viability of in vitro-cultured oocytes, 155 OGCs were allocated to 4 experimental replicates, and oocyte survival rate and maturation rate were quantified.

#### 2.2.3. Experiment 2: Investigation of the Effect of IGF-I on Granulosa Cell Apoptosis

After confirming the optimal supplemental concentration of IGF-I, granulosa cells were collected from OGCs that had been cultured for 14 days with the optimal IGF-I concentration and from the control group (without IGF-I treatment). Quantitative polymerase chain reaction (qPCR) was performed to detect the expression levels of apoptotic and anti-apoptotic genes, thereby clarifying the regulatory effect of IGF-I on the expression of these genes.

#### 2.2.4. Experiment 3: Study on the Effect of PFF in Culture Medium on Oocyte Growth and Development

Different concentrations (0, 2.5%, 5%, 10%; *v*/*v*) of PFF (not heat-inactivated) were added to the culture medium. Indicators such as oocyte growth, survival rate, and maturation rate were detected to explore whether the addition of PFF to the culture system could exert beneficial effects on oocyte growth. For the PFF treatment experiment, two sets of experiments were conducted with 6 replicates each: 231 OGCs were used to determine oocyte diameter and OGCs’ antrum formation rate, while 232 OGCs were utilized to evaluate oocyte survival and maturation rates.

### 2.3. Ovary Collection and Porcine Follicular Fluid Preparation

Porcine ovaries were acquired from a local slaughterhouse (Wanxin Meat Products Co., Ltd., Jilin, China). Donor pigs were in diestrus, and a total of 232 well-developed ovaries with corpora lutea and no signs of ovulation were selected for the experiment. The obtained ovaries were preserved in physiological saline at 37 °C, containing 100 IU/mL penicillin and 50 µg/mL streptomycin. The ovaries were subsequently shipped to the laboratory within a 2 h timeframe. PFF was aspirated from 3 to 6 mm follicles using a 10 mL sterile disposable syringe fitted with an 18-gauge hypodermic needle. After centrifugation at 1500× *g* at room temperature for 30 min to thoroughly remove cellular debris, avoiding interference with OGC culture, the PFF was filtered through 0.22 µm filter and stored at −20 °C.

### 2.4. OGC Collection and Culture

OGCs were collected and cultured according to the method previously described by Hashimoto et al., with some modifications [38]. Briefly, ovarian cortical tissue was sliced (<1 mm thickness) from the ovarian surface using a blade. Under a stereomicroscope, follicles with a diameter of 300–500 μm (part of early antral follicle [38]) were identified. Under a stereomicroscope, these follicles were punctured with a sterile 28-gauge needle to gently release intact OGCs. This procedure was performed in PBS supplemented with 3 mg/mL bovine serum albumin (BSA; Sigma-Aldrich LLC, St. Louis, MO, USA; A7638). Only healthy oocytes (with intact plasma membranes and uniform cytoplasm) surrounded by more than 3 layers of granulosa cells were used for the experiments. 4~5 OGCs were cultured in 50-μL droplets of culture medium under mineral oil in culture dish (Corning Life Science, Tewksbury, MA, USA; Falcon 353002), at 38.5 °C, 5% CO_2_, and high humidity. TCM 199 (Sigma, M4530) served as the base for the culture medium, which was further supplemented with 100 μg/mL ascorbic acid (Sigma, A4544), 1 μg/mL estradiol (Sigma, E8875), 10 μg/mL insulin (Sigma, I5523), 6.7 ng/mL sodium selenite (Sigma, S5261), 5.5 μg/mL transferrin (Sigma, T5391), 3 mg/mL BSA, 0.05 mg/mL gentamicin (Sigma, G1397), and 2% polyvinylpyrrolidone (Sigma, PVP360).

The experiments were designed for different groups of culture media supplemented with or without IGF-I (10, 50, or 100 ng/mL; Sigma, I3769) or PFF (2.5, 5, or 10% *v*/*v*). OGCs were cultured for 14 days. Half of the culture medium was replaced every 2 days. 

The ooplasm diameter and antrum-like structure formation in the OGCs were examined using a stereomicroscope (Nikon Corporation, Tokyo, Japan, SZM800N) with an imaging system (DS-Fi3). The diameter of the oocyte cytoplasm (excluding the zona pellucida and perivitelline space) was calculated as the average of the maximum distances along the horizontal and vertical axes. On Day 0, the oocyte diameter was measured by the same observer, followed by random grouping of OGCs for culture. After 14 days of culture, OGCs were treated with 0.2% hyaluronidase to remove the surrounding granulosa cells. Subsequently, the oocyte diameter was measured by an observer blinded to the experimental group assignments of the samples to eliminate potential subjective bias that could affect the experimental results.

### 2.5. Oocyte Maturation, Morphological Integrity Assessment

Following a 14-day culture period, maturation culture was performed on the OGCs. OGCs were cultured in 50-μL droplets of maturation medium covered with mineral oil in Petri dish (Falcon 3002). The maturation medium was TCM-199 supplemented with 0.57 mM L-cysteine (Sigma, C7352), 3.05 mM D-glucose (Sigma, G6152), 0.91 mM sodium pyruvate (Sigma, P4562), 0.5 µg/mL LH (Sigma, L5269), 0.5 µg/mL FSH (Sigma, F2293), 10 ng/mL epidermal growth factor (Sigma, E4127), 75 µg/mL penicillin (Sigma, PENK), 50 µg/mL streptomycin (Sigma, S6501), 0.05% (*v*/*v*) MEM vitamins (Sigma, M-6895) and 10% (*v*/*v*) porcine follicular fluid. After maturing for 22 h under conditions of 38.5 °C, 5% CO_2_ and saturated humidity, the culture was switched to maturation medium without FSH and LH for an additional 22 h maturation period (to enhance cytoplasmic maturation and cumulus expansion [39]). After in vitro maturation (IVM), the granulosa cells were removed from the oocytes using 0.2% hyaluronidase (Sigma, H3506). Under a stereomicroscope, oocyte morphological integrity and maturation were determined. Oocytes with dark and homogeneous cytoplasm, a distinct perivitelline space, and intact, non-deformed plasma membranes and zona pellucidae are considered morphologically intact and surviving. Oocytes were gently and comprehensively rotated in the maturation medium by a micropipette to examine all angles. The extrusion of the first polar body served as the criterion for classifying an oocyte as mature. An observer unaware of the samples’ experimental group assignments evaluated oocyte morphology and maturation to eliminate subjective bias affecting experimental results. In the IGF-I treatment experiment, 155 OGCs were used for maturation, with four replicates per experimental group. In the PFF treatment experiment, 232 OGCs were employed for maturation, and each experimental group was replicated six times.

### 2.6. Quantitative PCR

After 14 days of culture, granulosa cells were separated from oocytes using 0.2% hyaluronidase. The collected granulosa cells were then subjected to quantitative polymerase chain reaction (qPCR) analysis. Total RNA of granulosa cells derived from 50 OGCs in each group was isolated in accordance with the protocol provided by the Arcturus PicoPure RNA Isolation Kit (Thermo Fisher Scientific Baltics UAB, Vilnius, Lithuania, KIT0204). In each group, 100 ng of extracted RNA was used to synthesize complementary DNA (cDNA) by the PrimeScript RT reagent Kit with gDNA Eraser (Perfect Real Time) (Takara Bio Inc., Kusatsu, Shiga, Japan, RR047A). RT-qPCR was performed in a 20 µL reaction mix that included 2 µL cDNA template, 1 µL each of forward and reverse primers (10 pmol/µL), 10 µL TB Green^®^ Premix Ex Taq™ II (Takara, RR820A), and 6 µL nuclease-free water. Thermal cycling conditions started with an initial denaturation at 95 °C for 30 s, then 40 cycles of 95 °C for 5 s (denaturation) and 60 °C for 30 s (annealing). The qPCR assays were performed in 3 replicates. The 2^^(−ΔΔ*Ct*)^ method was applied to compute relative mRNA abundances of target genes. *GAPDH* (which has been demonstrated to be a stable internal reference gene for studies on porcine granulosa cells and oocytes [40,41,42]) was used as the internal reference gene. The sequences of the primers are presented in Table 1.

### 2.7. Statistical Analysis

All resulting data are expressed as the mean ± SEM. Statistical evaluations were performed using Statistical Product and Service Solutions (SPSS) 26.0 software (IBM, 233 South Wacker Drive, 11th Floor, Chicago, IL, USA). The normality of data distribution was verified using the Shapiro–Wilk test, and homogeneity of variances was assessed via Levene’s test. Data analysis on antrum-like structures formation, oocyte growth, survival rate and maturation rate were performed by One-way analysis of variance (ANOVA) to identify significant differences among the experimental groups. Afterwards, Duncan’s multiple comparisons test was conducted. Independent sample *t*-test was performed to analyze the qPCR data of granulosa cell apoptosis. Statistical significance was defined as a *p* value < 0.05.

## 3. Results

### 3.1. Formation of Antrum-like Structures of OGCs

**Figure 1:**
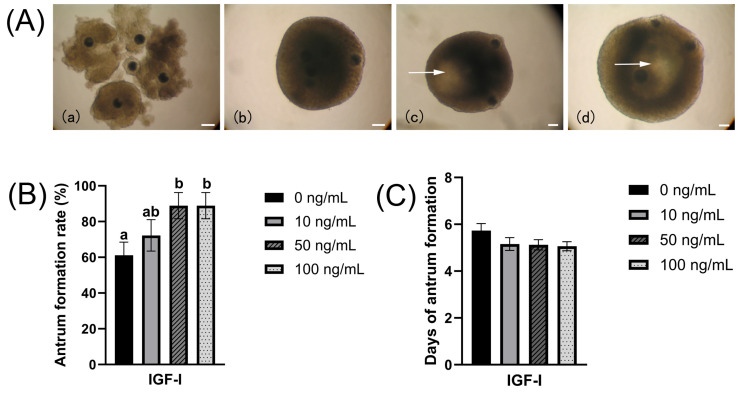
In vitro growth of oocyte-granulosa cell complexes (OGCs). (**A**) Morphological changes in OGCs during growth: (**a**) Freshly collected OGCs were designated as Day 0 of culture. (**b**) After being cultured for 3 days, the OGCs grew into spherical structures. (**c**) After being cultured for 7 days, the spherical structures increased in size, and antrum-like structures appeared inside the OGCs (as indicated by the arrow). (**d**) After the OGCs were cultured for 14 days, the antrum-like structures became larger. The scale bar represents 100 µm. (**B**) Antrum-like structure formation rate of OGCs; (**C**) The onset of antrum-like structure formation. Columns without a common letter above them indicate significant differences (*p* < 0.05), while columns sharing the same letter or having no letter indicate non-significant differences (*p* > 0.05).

In preliminary experiments, it was found that when OGCs were cultured for 14 days, the oocyte diameter, survival rate and maturation rate reached the optimal levels. IGF-I concentrations above 100 ng/mL resulted in a decrease in oocyte survival rate and maturation rate, while the addition of PFF failed to promote oocyte growth and survival (the survival rate even exhibited a decreasing trend). Therefore, in the present study, we selected 14 days of OGC culture, an IGF-I concentration range of 0–100 ng/mL, and a PFF concentration range of 0–10% as the experimental parameters for subsequent investigations. When OGCs were cultured with or without IGF-I, antrum-like structures (antrum) formed within the complexes (Figure 1A). These structures were morphologically analogous to follicular antrums. In the present study, the formation rate (Figure 1B) and the onset of antrum-like structures formation (Figure 1C) across all experimental groups were quantified. Statistical analysis revealed that, compared with that in the control group (61.11 ± 7.35), the antrum formation rate was significantly increased in the groups treated with 50 (88.89 ± 7.35) and 100 ng/mL IGF-I (88.89 ± 7.35) (Figure 1B, *p* < 0.05). These results indicate that culture with 50 ng/mL IGF-I can facilitate the formation of antrum-like structures in OGCs. With respect to the onset of antrum-like structure formation, no significant differences were observed among the experimental groups (Figure 1C) (0 ng/mL: 5.73 ± 0.30; 10 ng/mL: 5.15 ± 0.27; 50 ng/mL: 5.13 ± 0.22; 100 ng/mL: 5.06 ± 0.19, *p* > 0.05). These findings suggest that, regardless of its concentration, IGF-I does not accelerate the onset of antrum-like structure formation in OGCs.

### 3.2. In Vitro Growth of OGCs Cultured with IGF-I

To evaluate the effect of IGF-I on oocyte growth, oocyte diameters were measured. At the start of culture (Day 0), there was no significant difference in oocyte diameter among all groups (Table 2, *p* > 0.05). After 14 days, the oocyte diameter did not significantly differ between the 50 and 100 ng/mL IGF-I groups (Table 2, *p* > 0.05). However, both 50 and 100 ng/mL groups had significantly larger diameters than the control and 10 ng/mL groups (Table 2) (50 ng/mL, 100 ng/mL vs. 0 ng/mL, 10 ng/mL: 114.94 ± 0.58, 113.29 ± 0.50 vs. 108.77 ± 0.27, 109.83 ± 0.54, *p* < 0.05).

**Table 2:** The diameter growth of oocytes cultured with IGF-I.

IGF-I (ng/mL)	No. of OGCs (Replicates)	Diameter of Oocyte on Day 0 (µm, Mean ± SEM)	Diameter of Oocyte on Day 14 (µm, Mean ± SEM)
0	84 (9)	93.77 ± 0.16	108.77 ± 0.27 ^a^
10	85 (9)	93.92 ± 0.15	109.83 ± 0.54 ^a^
50	82 (9)	94.03 ± 0.16	114.94 ± 0.58 ^b^
100	84 (9)	93.76 ± 0.16	113.29 ± 0.50 ^b^

In the same column, values marked with different superscripts differ significantly (*p* < 0.05). Values with the same letter or without superscripts indicate no significant difference (*p* > 0.05). Table 3, Table 4 and Table 5 follow the same convention.

**Table 3:** Effect of IGF-I on the viability of oocyte cultured in vitro.

IGF-I (ng/mL)	No. of OGCs (Replicates)	Survival Rate No. (%, Mean ± SE)	Maturation Rate No. (%, Mean ± SE)
0 (control)	40 (4)	21 (52.50 ± 3.66)	10 (25.00 ± 3.27) ^a^
10	39 (4)	21 (54.38 ± 4.58)	11 (28.13 ± 3.53) ^ab^
50	37 (4)	23 (61.88 ± 3.78)	14 (38.13 ± 3.77) ^b^
100	39 (4)	22 (56.25 ± 4.61)	13 (33.13 ± 3.40) ^ab^

Survival rate = No. of surviving oocytes after 14 days of culture and 2 days of maturation/Total No. of OGCs in culture. Maturation rate = No. of mature oocytes/Total No. of OGCs in culture.

**Table 4:** Diameter growth of oocyte cultured with porcine follicular fluid (PFF).

PFF	No. of OGCs (Replicates)	Diameter of Oocyte on Day 0 (µm, Mean ± SEM)	Diameter of Oocyte on Day 14 (µm, Mean ± SEM)
0%	60 (6)	93.23 ± 0.21	108.92 ± 0.64
2.5%	57 (6)	92.99 ± 0.15	108.49 ± 0.37
5%	56 (6)	93.12 ± 0.18	108.77 ± 0.62
10%	58 (6)	93.42 ± 0.13	108.08 ± 0.65

**Table 5:** Viability of oocyte cultured with porcine follicular fluid.

PFF	No. of OGCs (Replicates)	Survival Rate No. (%, Mean ± SEM)	Maturation Rate No. (%, Mean ± SEM)
0	57 (6)	31 (54.58 ± 4.67) ^a^	16 (28.33 ± 4.00)
2.5%	59 (6)	23 (39.17 ± 3.98) ^b^	12 (20.42 ± 4.28)
5%	58 (6)	22 (37.92 ± 4.15) ^b^	11 (19.17 ± 3.07)
10%	58 (6)	24 (41.25 ± 4.18) ^b^	12 (20.83 ± 3.53)

Survival rate = No. of surviving oocytes after 14 days of culture and 2 days of maturation/Total No. of OGCs in culture. Maturation rate = No. of mature oocytes/Total No. of OGCs in culture.

### 3.3. The Viability of Oocytes Cultured In Vitro

**Figure 2:**
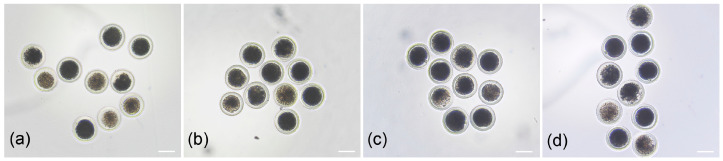
Morphological comparison of oocytes after 14-day culture and subsequent maturation. (**a**) Oocytes in control group; (**b**) Oocytes in 10 ng/mL treatment group; (**c**) Oocytes in 50 ng/mL group; (**d**) oocytes in 100 ng/mL group. The scale bar represents 100 µm.

To investigate the effect of IGF-I on the viability of oocytes following in vitro growth, we performed morphological assessment and maturation rate detection on oocytes after growth and maturation. As shown in Figure 2, the 50 ng/mL IGF-I treatment group exhibited superior oocyte morphology compared to the control group. However, statistical analysis of oocyte survival rate (Table 3) revealed that, although the survival rate in the 50 ng/mL treatment group exhibited a slight increase compared with the other groups, no significant difference was observed among all groups (*p* > 0.05). After maturation, results indicated that the oocyte maturation rate was significantly greater in the 50 ng/mL group than in the control group (50 ng/mL vs. 0 ng/mL: 38.13 ± 3.77 vs. 25.00 ± 3.27, *p* < 0.05). In contrast, there was no significant change in the maturation rate among the 0, 10, and 100 ng/mL groups (*p* > 0.05).

### 3.4. Influence of IGF-I Treatment on Granulosa Cell Apoptosis

**Figure 3:**
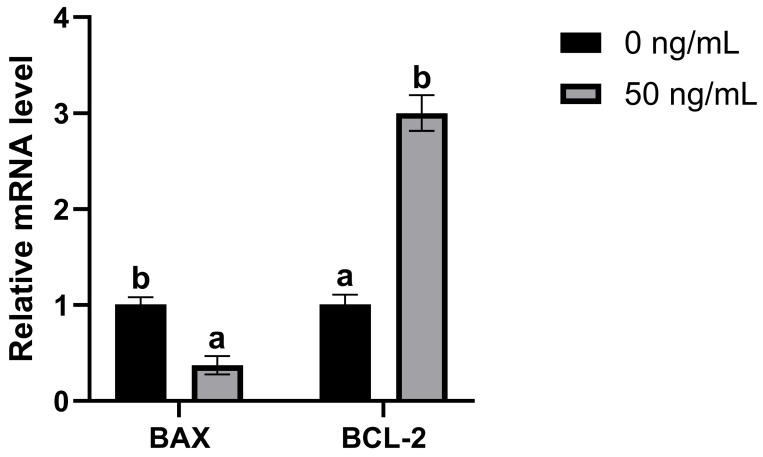
Relative transcript levels of *BAX* and *BCL-2* of granulosa cells in oocyte-granulosa cell complexes with or without IGF-I treatment. 0 ng/mL represents the control group, and 50 ng/mL represents the IGF-I treatment group. *GAPDH* served as a reference gene. Different letters above columns denote significant differences (*p* < 0.05) and the same letter indicates no significant difference (*p* > 0.05) in each gene.

Based on the previous results, 50 ng/mL was identified as the most effective concentration. Consequently, comparative detection of apoptotic and antiapoptotic genes was performed between the 50 ng/mL group and the control group to investigate whether IGF-I promotes oocyte development by regulating granulosa cell apoptosis. Compared with the control group, IGF-I treatment reduced the expression of the apoptotic gene *BAX*. Additionally, IGF-I increased the mRNA expression of *BCL-2* (an antiapoptotic factor) (Figure 3, *p* < 0.05).

### 3.5. Changes in the Growth Pattern of OGCs Cultured in Medium Supplemented with PFF

**Figure 4:**
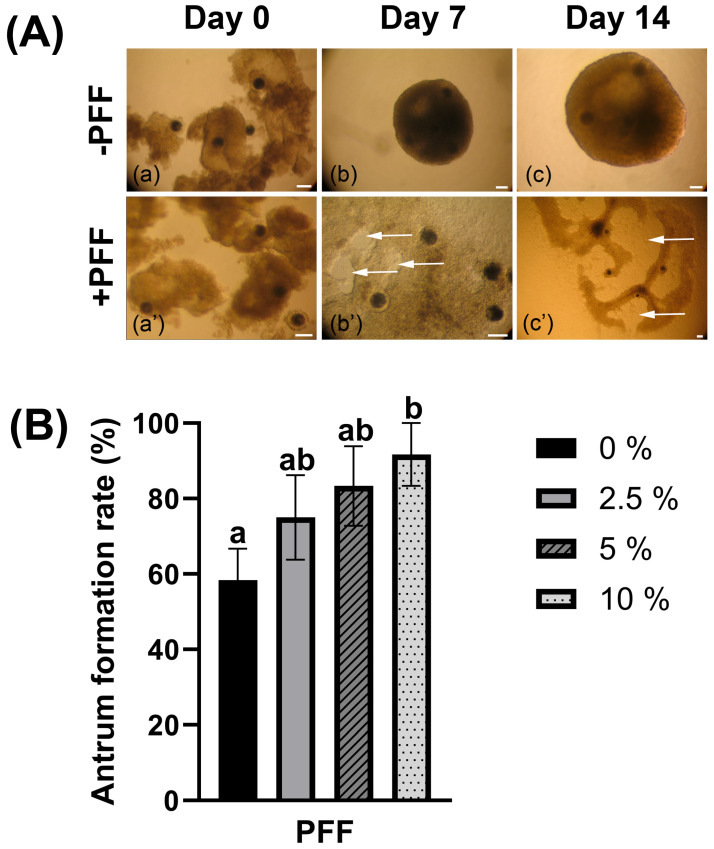
In vitro growth of oocyte-granulosa cell complexes (OGCs) treated with or without porcine follicle fluid (PFF). (**A**) Morphological changes in OGCs cultured without (−PFF) or with PFF (+PFF): (**a**,**a’**) Fresh OGCs were collected, designated as Day 0 of culture; (**b**,**b’**) OGCs were cultured for 7 days, and an enclosed antrum-like structure appeared inside the OGCs cultured without PFF. In contrast, OGCs supplemented with PFF formed open antrum-like structures (indicated by the arrow). (**c**,**c’**) After 14 days of culture, the sizes of antrum-like structures within the OGCs increased in both the −PFF and the +PFF groups. A 100 μm scale bar is provided in each image. (**B**) Antrum-like structures formation (antrum formation) rate. Columns without a common letter above them indicate significant differences (*p* < 0.05), while columns sharing the same letter indicate non-significant differences (*p* > 0.05).

OGCs cultured with PFF induced a distinct change in the growth pattern of OGCs (see Figure 4A). Unlike the spheroid-like growth morphology observed in the PFF-free (−PFF) control group, the granulosa cells in the PFF-supplemented group (+PFF) exhibited adherent growth characteristics. Consequently, the oocytes were left exposed to the culture medium and failed to be encapsulated by the surrounding granulosa cells. When cultured for 5~7 days, the OGCs treated with PFF formed antrum-like structures; however, these structures were found to be open-chambered. Such structures are incapable of establishing a functional microenvironment that facilitates bidirectional communication and mutual support between oocytes and granulosa cells. The effect of porcine follicular fluid on OGC growth was examined in the present study. The formation rate of antrum-like structures in OGCs and the diameter of oocytes were assessed in groups supplemented with or without PFF. While the addition of 10% PFF increased the proportion of open-chambered antrum-like structure formation (Figure 4B) (10% vs. 0: 91.67 ± 8.33 vs. 58.33 ± 8.33, *p* < 0.05), it failed to increase oocyte growth (Table 4, *p* > 0.05). The results of the oocyte survival assays indicated that the survival rate of the oocytes cultured with PFF was lower than in the control group (Table 5) (2.5%, 5%, 10% vs. 0: 39.17 ± 3.98, 37.92 ± 4.15, 41.25 ± 4.18 vs. 54.58 ± 4.67, *p* < 0.05). None of the PFF treatment groups exhibited an improvement in oocyte maturation rate (Table 5, *p* > 0.05).

## 4. Discussion

IGF-I is a key member of the insulin-like growth factor family [43] and acts as an essential mediator of growth hormone function. It plays a crucial role in cell differentiation, proliferation, and embryonic development [44,45,46]. During ovarian folliculogenesis, IGF-I regulates the proliferation, differentiation, and apoptosis of granulosa cells [22,47,48,49].

Crosstalk between oocytes and granulosa cells is essential for proper development of oocytes, as numerous factors secreted by both cell types mutually influence each other [50,51]. During follicular development, a follicular antrum is formed, which secretes follicular fluid. This configuration allows for the capture of factors secreted by granulosa cells that surround the oocyte, thereby creating a microenvironment conducive to oocyte growth. During OGC culture, we observed that OGCs formed closed antrum-like structures similar to follicle as granulosa cells proliferate. This result is consistent with observations reported by Hashimoto et al. [38]. While this antrum-like structure has not yet been confirmed to be identical to the follicular antrum, it appears closely associated with oocyte development [52,53] by promoting growth [54]. The data from this study revealed that the addition of 50 ng/mL IGF-I significantly increased the rate of antrum-like structure formation in OGCs. This might be attributed to the ability of IGF-I to promote granulosa cell proliferation and differentiation at an appropriate concentration. This effect thereby induces the formation of antrum-like structures in a greater number of OGCs. The antrum-like structures recapitulate the in vivo follicular microenvironment, serving as a key driver of oocyte growth and maturation. This structure concentrates paracrine factors (e.g., IGF-I, EGF) secreted by granulosa cells, creating a locally enriched milieu that facilitates nutrient delivery and metabolic waste removal for oocytes [51,55]. The antrum-like structure can restrict the movement and diffusion of these factors, keeping them constantly enclosed by the oocytes, which benefits oocyte growth. It also enhances oocyte-granulosa cell crosstalk, promoting meiotic progression and cytoplasmic maturation by amplifying signaling pathways critical for oocyte competence. This closed structure can further encase and protect oocytes, mitigating external oxidative stress-induced damage. Collectively, these findings highlight the antrum-like structure’s role in mimicking physiological follicular function, underscoring its importance in optimizing in vitro growth systems for porcine oocytes.

Sugiyama et al. demonstrated that the number of granulosa cells encircling the oocyte is associated with oocyte developmental competence [56]. The number of granulosa cells affects lipids within the oocyte, which serve as a crucial indicator of oocyte developmental capacity [57]. Therefore, an adequate number of granulosa cells can promote oocyte growth and development. In granulosa cells, IGF-I binds to IGF-IR and activates PI3K, which further phosphorylates Akt. This activation promotes cell cycle progression from G0/G1 to S phase to boost proliferation [48]. Our results indicated that 50 ng/mL IGF-I significantly promoted oocyte growth. This might be because IGF-I can trigger mitosis in the granulosa cells surrounding the oocytes according to the PI3K/Akt pathway, leading to their substantial proliferation and the secretion of growth factors, which in turn ensure normal oocyte development. However, higher concentrations of IGF-I (e.g., 100 ng/mL in our study) may not further improve oocyte quality. IGF-I exerts a dose-dependent effect on follicular development, and high concentrations may induce excessive follicular activation and atresia, thereby accelerating the depletion of ovarian reserve [58,59]. The long-term effects of IGF-I on oocyte epigenetic status and subsequent embryonic development require further verification. The applicability of this concentration to other species (e.g., cattle, sheep) needs to be tested in future studies.

IGF-I can inhibit granulosa cell apoptosis [60,61]. Following activation of the PI3K/Akt pathway by IGF-I, phosphorylated Akt exerts anti-apoptotic effects. IGF-I modulates the antiapoptotic protein *BCL-2* through a transcriptional mechanism, activating the nuclear transcription factor cAMP-response element-binding protein to induce *BCL-2* expression [62]. Consequently, we hypothesized that incorporating IGF-I into the culture medium may facilitate oocyte growth by suppressing granulosa cell apoptosis. To validate this hypothesis, qPCR analysis of granulosa cells from 14-day-cultured OGCs showed significantly reduced mRNA expression of pro-apoptotic *BAX* and increased antiapoptotic *BCL-2* mRNA levels in the IGF-I-treated group. This observation may be attributed to IGF-I at an optimal concentration activating the PI3K/Akt pathway in granulosa cells, which inhibits pro-apoptotic *BAX* activity and upregulates anti-apoptotic *BCL-2* expression, thereby increasing the *BCL-2*/*BAX* ratio to suppress apoptosis. *BCL-2* expression can suppress the generation of mitochondrial reactive oxygen species (ROS). In turn, this reduction inhibits apoptosis [63]. Therefore, adding an appropriate amount of IGF-I during OGC culture may facilitate oocyte growth through a series of regulatory effects. Specifically, it can modulate the balance of *BAX* and *BCL-2* in granulosa cells. Furthermore, it may inhibit ROS production in granulosa cells, preventing apoptosis. In this way, it reduces the risk of OGCs undergoing atresia.

Follicular growth and maturation are accompanied by dynamic changes in follicular fluid components, and 3–6 mm follicles represent a critical transitional stage between early antral and preovulatory follicles—characterized by peak concentrations of growth factors, cytokines, and nutrients that are essential for oocyte-granulosa cell crosstalk and oocyte competence acquisition [33,64,65]. These factors from larger transitional follicles can exert beneficial effects to promote early oocyte development, such as enhancing granulosa cell proliferation and oocyte competence [31,66]. Our findings revealed that OGCs cultured with porcine follicular fluid (+PFF group) did not significantly enhance oocyte growth, as anticipated. Compared with the PFF-free control group (−PFF group), oocyte diameter in the +PFF group did not increase significantly (*p* > 0.05). FF is not a uniform medium but a dynamic cocktail whose components vary with follicle size, developmental stage, and individual physiology. Our PFF was isolated from 3 to 6 mm antral follicles, which contain fully grown oocytes. Follicular fluid at this stage not only contains growth-promoting factors for oocytes such as IGF-I, EGF, and GH, but also inhibitory factors such as Anti-Müllerian hormone (AMH) [67], which may not be conducive to the growth of oocytes derived from early antral follicles. Follicle-stimulating hormone (FSH) drives follicular growth and granulosa cell proliferation by upregulating *CYP19*, with E_2_ production correlating with follicular development [68]. Montani et al. found that AMH suppresses FSH-induced *CYP19* gene/protein expression, potentially modulating granulosa cell activity and folliculogenesis via E_2_ synthesis [69]. To date, most studies on PFF have focused on oocyte maturation, with treatment limited to within 44 h of porcine oocyte maturation [33,36]. In contrast, our experiment requires PFF supplementation during 14 days of OGC culture, which may exacerbate the accumulation of AMH and thereby impair OGC development.

Notably, the OGCs in the −PFF group exhibited a spherical growth pattern. In contrast, granulosa cells of OGCs in the +PFF group flattened and spread on the surface of the culture dish. After approximately 5 days of culture, the OGCs in the +PFF group formed “open” antrum-like structures. Unlike the closed antrum structures developed by OGCs in the -PFF group, the antrum-like structures in the +PFF group failed to enclose the oocytes, thus failing to provide a specialized microenvironment necessary for oocyte growth. Without the support of this closed microenvironment, the bidirectional interactions between granulosa cells and oocytes were diminished; this may explain why PFF supplementation failed to promote oocyte growth and development. Laminin, fibronectin, collagen, and proteoglycans are present in the follicular fluid. They are among the components of the extracellular matrix (ECM) [32,70]. The observation that PFF promotes adherent granulosa cell growth is indeed an unexpected and notable finding, which may be attributed to the synergistic action of ECM components, cell surface receptors, and exosomal cargos in PFF. PFF is enriched with ECM molecules such as fibronectin—a key mediator of granulosa cell cytodifferentiation and adhesion—and these components can form a provisional matrix that supports cell attachment [71,72]. This process is likely mediated by integrins (e.g., integrin α6) expressed on porcine granulosa cells, as integrin-ECM interactions are known to regulate granulosa cell adhesion and folliculogenesis [73]. Consistent with this, recent studies on porcine granulosa cells have confirmed that ECM-integrin signaling crosstalk is closely involved in regulating cell adhesion and migration, with exosomal components in follicular fluid further modulating these adhesive processes [74]. These ECM molecules, synergized with exosomes in PFF, may bind to integrin receptors expressed on the surface of granulosa cell membranes, triggering granulosa cells to extend pseudopodia, anchor to the culture substrate, and spread. Subsequent migration of these spread granulosa cells could then lead to the generation of the open antrum-like structures observed in the +PFF group [70,75]. However, this remains a speculative interpretation; the specific molecular mechanisms governing the adherent growth of granulosa cells in the presence of PFF require further investigation. In the present study, only a few doses of PFF were tested, and the results correspond only to the tested doses. The potential of follicular fluid from follicles of other diameters (e.g., 1–3 mm) or other applied doses (e.g., 15–20%) to support the in vitro growth of oocytes remains to be further experimentally validated. The content of ECM components in PFF can also be determined to identify the specific molecules that mediate granulosa cell adhesion.

Currently, in vitro oocyte culture and growth remain underdeveloped with relatively low efficiency. Establishing a mature technology for in vitro oocyte growth could expand the sources of oocytes required for assisted reproductive technologies. Our research has provided insights and data supporting the advancement of in vitro oocyte growth and culture technology. This lays a foundation for the “large-scale acquisition of high-quality oocytes” in porcine reproduction and breeding, and its application scenarios can be further expanded by integrating cryopreservation technology. In this study, we identified 50 ng/mL as the optimal IGF-I concentration that specifically enhances porcine OGCs’ antrum formation, oocyte growth, and maturation, with clear regulatory effects on apoptotic genes and anti-apoptotic factor (*BAX*/*BCL-2*)—this dose-dependent specificity and mechanism in porcine OGCs have not been fully elucidated before. Unlike most studies reporting beneficial effects of follicular fluid, our results demonstrate that PFF at tested concentrations (2.5%, 5%, 10%) induces abnormal adherent growth of granulosa cells and fails to promote oocyte development, providing critical insights into the limitations of PFF supplementation in porcine OGC culture. This may be attributed to the limitations associated with PFF concentration or the choice of follicle size; thus, these factors warrant further investigation in subsequent studies.

There are several limitations in this study. First, we used *GAPDH* as the sole reference gene for qPCR normalization, and future studies could include additional stable reference genes to enhance data robustness. Second, our analysis focused on a limited gene panel related to apoptosis and lacked protein-level validation. Finally, we only selected PFF from 3 to 6 mm follicles and tested a few concentrations, which is insufficient to fully elucidate PFF’s regulatory effects on OGC culture. Other concentrations of PFF and selection of follicle sizes remain unexplored, and subsequent research could explore these additional factors to investigate their effects on in vitro oocyte growth. Future studies should focus on exploring more in-depth mechanisms, such as mitochondrial membrane potential, spindle morphology in MII oocytes under IGF-I regulation, reactive oxygen species inhibition and apoptosis pathway regulation. Expanding the gene set to include those involved in key signaling pathways (e.g., PI3K/AKT, MAPK) or extracellular matrix remodeling would provide a more comprehensive understanding of PFF and IGF-I’s regulatory networks.

## 5. Conclusions

In conclusion, our study identifies 50 ng/mL IGF-I as the optimal dose for enhancing the in vitro development of OGCs derived from EAFs, with key beneficial effects including reduced granulosa cells’ apoptosis, promoted formation of functional antrum-like structures, and improved oocyte diameter growth and maturation efficiency. Notably, higher concentrations of IGF-I (100 ng/mL) do not yield additional benefits, indicating a dose-dependent regulatory effect of IGF-I on porcine EAF-derived oocytes. In contrast, PFF at the tested concentrations induces adherent and spreading growth of granulosa cells, leading to open antrum-like structures that fail to establish a supportive microenvironment for oocyte development. The mechanism by which PFF regulates the adherent and spreading growth of granulosa cells remains unclear. Collectively, these findings underscore the critical role of IGF-I dose optimization and the need to refine PFF-based culture strategies (e.g., concentration adjustment, follicle size selection) to improve the in vitro growth system for porcine EAF-derived oocytes, providing practical insights for advancing animal reproductive biotechnology.

## Figures and Tables

**Table 1:** Primer sequences.

Primer	Sequence	Product Size (Base Pairs)	GenBank Accession Number
*BAX*	F: AGCTGAGCGAGTGTCTCAAG	95	XM_003355975.2
	R: AGAAGAGACCACTCCTGGGT		
*BCL-2*	F: GAACTGGGGGAGGATTGTGG	164	XM_003121700.3
	R: CATCCCAGCCTCCGTTATCC		
*GAPDH*	F: GACCCCTTCATTGACCTCCA	131	NM_001206359.1
	R: TGGAAGATGGTGATGGCCTT		

## Data Availability

The raw data supporting the conclusions of this article will be made available by the authors on request.
